# Correction to: Bone mesenchymal stem cell-derived exosomal microRNA-29b-3p prevents hypoxic-ischemic injury in rat brain by activating the PTEN-mediated Akt signaling pathway

**DOI:** 10.1186/s12974-020-01872-8

**Published:** 2020-07-07

**Authors:** Kun Hou, Guichen Li, Jinchuan Zhao, Baofeng Xu, Yang Zhang, Jinlu Yu, Kan Xu

**Affiliations:** 1 Department of Neurosurgery, The First Hospital of Jilin University, Changchun, 130021 P.R. China; 2 Department of Neurology, The First Hospital of Jilin University, Changchun, 130021 P.R. China

**Correction to: J Neuroinflammation (2020) 17:46**

**https://doi.org/10.1186/s12974-020-1725-8**

Following publication of the original article [1], the authors noticed that the images in Fig. 7j were mistakenly selected, which were the images in Fig. 1g. Now, the authors provide Fig. 7 with all the confirmed correct images.

**Fig. 7:**
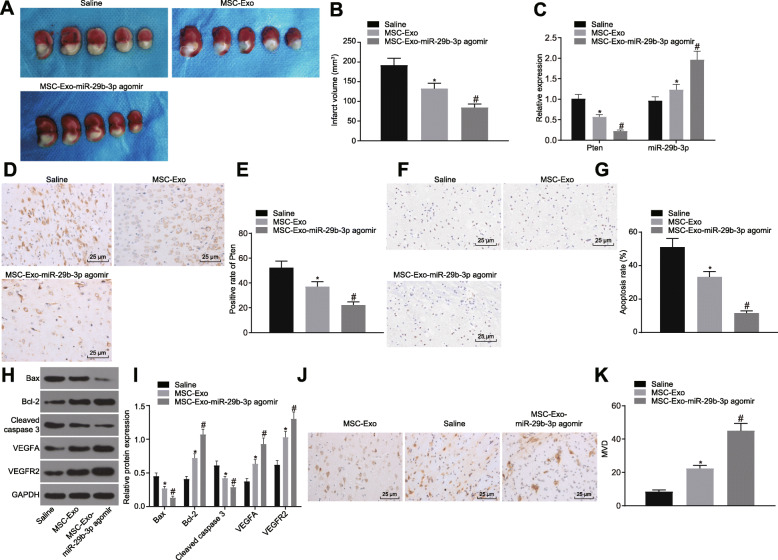
Exosomal miR-29b-3p from BMSCs ameliorates brain injury in MCAO rats. The brain of MCAO rats was injected with exosomes from untreated BMSCs or miR-29b-3p agomir over-expressed BMSCs. **a**, **b** The volume of brain injury shown by TTC staining. **c** The mRNA expression of PTEN and miR-29b-3p expression determined by RT-qPCR. **d**, **e** The protein expression of PTEN shown by immunohistochemistry staining (scale bar = 50 μm). **f**, **g** The apoptosis shown by TUNEL staining (scale bar = 50 μm). **h**, **i** The protein expression of apoptosis and angiogenesis-related genes measured by western blot analysis. **j**, **k** The immunohistochemical staining of CD31 and MVD in brain tissues (scale bar = 25 μm). *p < 0.05, vs. the saline group. #*p* < 0.05, vs. the MSCs-Exo group. The measurement data are expressed as mean ± standard deviation. The comparison among multiple groups was conducted using one-way analysis of variance, followed by Turkey’s post hoc test. The experiment was repeated three times, with *n* = 7 in each group
